# Customer engagement design during the COVID 19 pandemic, mutual trust and intelligent automation: a conceptual perspective

**DOI:** 10.1186/s13731-022-00222-7

**Published:** 2022-03-05

**Authors:** Abdullah Abdulmohsen Alfalih

**Affiliations:** Department of Business Administration, College of Business Administration, Majmaah University, Al-Majmaah, 11952 Saudi Arabia

**Keywords:** Customer engagement (CE), Intelligent automation (IA), Mutual trust, Co-construction, Artificial intelligence (AI)

## Abstract

The adoption and use of artificial intelligence, and the application of this concept through the development and implementation of intelligent automation is not considered simply as an option, but rather as an obligation in current times, due to the considerable change caused by the COVID 19 pandemic and responses to it. This study is an attempt to more thoroughly understand and clarify how the adoption of such intelligent automation can work to improve customer engagement in the food and restaurant domain. To attend to this objective, a theoretical framework is developed and tested based on an integrative approach of determinants of customer engagement through artificial intelligence to increase trust levels when intelligent automation is used. This paper will contribute to the construction of a matrix of customer engagement based on the different steps identified in the customer engagement cycle, and build a co-constructive and dynamic model of customer engagement in relation to mutual’ trust and intelligent automation.

## Introduction

The most important recommendation in order to avoid COVID 19 is social distancing to decrease spread of the infection. In this case, artificial intelligence (AI) seems to be the immediate and most adequate solution to the need to maintain activities. AI replaces human workers toward the automatization of work by intelligent automation (IA). In this sense, AI is applied, and IA is used to work safely.

DeCanio (2016) defines AI as a suite of technologies which can exceed human capabilities, resolve problems and surpass human limits, reporting that AI covers many fields and can be appreciated through a large number of applications. For the purposes of this study, it is considered that AI is related to technologies such as robots and computer vision.

During the crisis of COVID 19, AI has been widely used to resolve problems caused by the unavailability of workers. It is used in health to provide solutions, assistance and treatments, and also to understand transmission (Bullock et al., 2020). AI is also adopted in customer service to replace human workers (Howard & Borenstein, 2020). This process of automatization can be applied in different forms, and is called intelligent automation (Coombs et al., 2020). Coombs et al. (2020) consider that IA can improve ways of learning, adapt and improve the automatization process of any task to replace humans. This supposes that during its adoption, an adequate process of learning is required to reach sufficient understanding, and then, that the knowledge extracted will be applied to actual situation and context in which it was developed. Progress can then be generated through the definition of a new way to accomplish the given task with increased efficiency. So, in this process, knowledge, know-how and know-how-to-be are developed.

In the current case, this process of automatization is adopted to determine how to increase, develop and ensure customer engagement (CE) in the domain of food and restaurants. Clearly, questions related to the quality of food also regain importance in the situation imposed by COVID 19. Online customer behavior and customer engagement behavior seem to be significant in this new situation. Further, a long-term relationship is critical in the service sector (Gurviez, 1999), and now by social restrictions caused by COVID 19. This conception of CE important, being related at the same time to the consumer in terms of satisfaction and security, especially if talking about health, and to the service provider in terms of rentability, continuity and survival, especially considering the economic difficulties encountered by the majority of businesses at the current time.

To demystify this interaction and be able to build a long-term relationship, a relationship marketing concept is taken as a reference which represents a set of tools used to generate a positive attitude toward a company or brand through an interactive and personalized relationship (Lendrevie et al., 2006). However, it is supposed within this field that interactivity can be covered by AI, and the concept of IA as responding to the needs of the customer, which can improve satisfaction and trust. This objective exceeds simple action/reaction over a short time, and seeks a long-time relation, maintained for as long as possible through the development of customer engagement (CE) in the service sector.

Customer engagement is a strategy which aims to build a sustainable relationship between organizations and customers (Kumar et al., 2010) in order to increase corporate performance (CP) (Kumar & Pansari, 2016) and acquire a sustainable competitive advantage (SCA) (Kumar & Pansari, 2016). This concept of engagement is related in general to psychology and educational fields (Berkman et al., 2000). Customer engagement is seen to be important, but still there is no consensus on its definition, determinants, factors or measures (Katagiri, 2020). This first observation constitutes one of the main objectives of this paper, which is related to this concept, in order to define it as well as identify its determinants. The literature review reveals that there are numerous and heterogeneous factors which can stimulate CE, such as psychological factors related to customer satisfaction (Kumar et al., 2019; So et al., 2014), in addition to trust (van Doorn et al., 2010), commitment (So et al., 2014) and satisfaction, added to emotional attachment (Kumar et al., 2019). Other researchers discuss behavioral factors such as resource integration and knowledge sharing (Hollebeek et al., 2019).

Another group considers that CE is directly related to organizational factors like employee engagement, which can contribute to service quality (Kumar & Pansari, 2016). However, a recent approach considers that CE development is related to some external factors (Kumar et al., 2019; van Doorn et al., 2010).

These brief analyses demonstrate the existence of some direct and indirect influences on CE or what could be called mutuality. However, it is still unclear what kind of factors related to the mutuality aspect can be used to understand CE.

Patterson et al. (2012) and So et al. (2015) present CE as a crucial dimension for service loyalty. Many researchers have tried to explain and define strategies for CE through various sets of factors. Enginkaya and Esen (2014), in a study on online customer engagement, demonstrate that trust and commitment can stimulate CE. Hollebeek (2009) presents the same factors related to trust and commitment, in addition to satisfaction and loyalty. In his study, he identifies a different aspect of the notion of engagement; context, objects, phrases, dimensions and levels. Other researchers, such as Wirtz et al. (2013), consider that CE depends on the development of brand communities. Bolton (2011) developed a model related to challenges and opportunities in developing CE which measures and manages CE in relation to trust, loyalty, customer value, satisfaction and quality. Wei et al. (2013) present another conception of CE determinants according to which the relative importance of factors depends on the objectives and perceptions of the customer compared to the nature and importance of motivational drivers. This suggests that an adequate research model in this case must consider this dynamic aspect. Research by So (2014) confirms this dynamic aspect, because it demonstrates that CE positively affects brand loyalty. Based on this analysis, and in line with the literature review, an important variable closely related to this theme is identified, which is trust.

Eiglier and Langeard (1994) show the importance of trust for the service industry and businesses through the development of a social dimension of the relationship. It is considered a crucial variable for the exchange which can explain partnership behavior (Cheng et al., 2016; Su et al., 2014). Thus, the number of research studies related to trust, the importance of this factor and its determinants have increased along with the interest of practitioners in this concept (Anderson & Narus, 1990; Cheng et al., 2016; Dwyer et al., 1987). However, Simon (2007) argues that despite the great number of works in the literature related to this concept, there is no consensus about its nature.

Independently of its importance and definitions, and due to the special conditions in which this research takes place, the role of trust is amplified. Poon et al. (2017) demonstrate that trust can reduce risk in a turbulent environment which seems to be dynamic and complex, added to the uncertainty imposed by the new normal caused by the COVID 19 pandemic.

Through this conceptual paper, a dynamic, exhaustive and integrative model of CE development is developed, which can be used or tested on future research. The main objective was to identify links between CE, artificial intelligence and trust (from the two parties) as the main determinant of CE. In other words, this will be a co-constructed framework between customer and service provider. For this, CE and its related concepts must be defined, as well as its determinants as added to trust (mutual). A matrix for CE will also be established, and can orient the decision-maker in line with the phases of the CE cycle.

## Literature review

### Customer engagement

Sashi (2012) relates engaged customers to a partnership or collaboration with sellers, to increase the satisfaction of their needs as shared with other customers. Added to this, he insists on the role of social media as a facilitator of this interaction based on commitment and trust.

To understand how CE is constructed, six different stages defined by Sashi (2012) which are integrated into the CE cycle are adopted here: first, connection is reported, as a prerequisite to define relationships and begin the exchange. Then, interaction occurs to collect information with which to create value: creation and extraction (Prahalad & Ramaswamy, 2004). The majority of researchers agree that information technology is determinant at this stage, because it facilitates the exchange of information (Hanssen & Faegri, 2006; Sawhney et al., 2005). Next is a satisfaction which represents the results of interaction, and if satisfaction is achieved, customer and seller define a new relationship of engagement. At this stage, satisfaction is not a final objective, but an intermediate element in progressing and achieving the strategic goals of the organization (Mittal & Kamakura, 2001). This means that satisfaction can translate into a progressive construct which is built and co-created until a specific and required level is reached. Firat and Dholakia (2006) present empowerment as a final objective of marketing because it facilitates the definition of a specific partnership between customer and seller, allowing the mutual construction of customer desires and developed products. In other words, satisfaction is necessary for engagement, but is not in itself enough.

After satisfaction, retention is represented by a long-term relationship between customer and seller. Gustafsson et al. (2005) conclude that the interdependence or effect of some determinants on retention is still unclear. For example, they demonstrate that satisfaction positively affects retention, but that the effect of commitment differs from one type to another. Affective commitment does not affect retention, but calculative commitment has a positive effect on retention in this case. This observation confirms that the process of CE is a reflexive cycle: the customer here is not a passive actor but an active and reactive actor, and this explains the conclusions drawn here about the co-construction process of CE.

Retention is completed here by commitment, which leads to loyalty if a calculative dimension of commitment is developed. Commitment here is appreciated through two dimensions: affective and calculative. The affective dimension is related to trust and reciprocity, but the calculative dimension depends on the rationality of choice (Gustafsson et al., 2005).

Advocacy follows commitment, and is related to both satisfaction and loyalty (Sashi, 2012). At this stage, both calculative commitment and affective commitment are interrelated (Harrison-Walker, 2001) and the customers who are advocates are those who are connected and interacting with the seller. Reciprocity is required here too: in this sense, the company which advocates for the customer will receive and guarantee trust and loyalty (Urban, 2004). This exchange in terms of advocacy is highly important and is determinant of the future valuable and successful relationship between seller and customer (Nordin, 2009).

Finally, in the engagement which occurs when a satisfied and loyal customer communicates their opinion and feelings through interaction through an artificial intelligence channel, and both sides of the partnership are advocates access to engagement is achieved (Sashi, 2012).

Based on this presentation related to the customer engagement cycle, we can conclude that the definition of CE depends on the nature and the strength of the relationship between customer and sellers or service provider. Emotional bonds are determinant, as are relational bonds. There is harmony between the two partners, and cooperation, which can bring benefits for both of them. The customer must be able to communicate their needs and the service provider must be able to respond and maximize the satisfaction of these needs. A customer here is at once a consumer and provider of information. This process of interaction and communication can be facilitated by AI in different ways and on different levels. The use of IA can make such information valid and reliable in real time.

Another emergent concept is mutual trust. This concept suggests that the customer believes that the service provider will be able to respond exactly as expected. On the other hand, the service provider must trust the customer to communicate dissatisfaction if it exists, in order to make adequate modifications or progressions. This kind of trust must define the corrective behavior which can be beneficial for each party. If CE is generated, a long-term relationship is generated.

Beyond this analysis related to the dynamic and the co-constructive approach, CE as a concept can be considered through four main perspectives: social, emotional, cognitive and behavioral investment in the firm or brand (Hollebeek et al., 2019; Kumar et al., 2019) based on an interactive view. This means that there is a bi-directional effect between consumer and firm if considering CE based on a communicational process, or at least an exchange of the right information at the right time. In this conception, AI and the use of IA regains importance. The next section will discuss how the use of IA can reinforce CE.

### Artificial intelligence, intelligent automation and customer engagement

Intelligent automation brings together automation and artificial intelligence (Accenture, 2016) to allow the digitalization of manufacturing and facilitate changes through the adoption of new technology which permits autonomous interaction with people and tasks, affecting the value chain (European Parliament, 2016).

Currently, with the new context and changes caused by COVID 19, the majority of people recognize the importance of the use of IA instead of human interaction: however, some consumers continue to prefer human interaction rather than a fully automized experience (Thomas, 2020). It is suggested here that consumer preferences must be changed, and the use of IA encouraged, to prevent spread of infection. In other words, the aim is to change consumer behavior and perceptions of the use of AI.

The integration of IA within the CE framework defined here is seen as related to its features and the data characteristics generated through the IA. To understand how this can be useful and definitive for the current context features and data characteristics will be detailed, to construct a clear conception of the foundation of the model.

Tiago et al. (2019) present some specific features of IA, such as: interoperability, related to the development of standards and protocols to exchange data and exploit information to produce results; virtualization, which represents a reproduction of the physical environment (Liu et al., 2018); visualization, which defines the interface between the user and the system; traceability or tracking of resources in real-time and its integral life cycle; decentralization, to make decision-making more flexible; real-time, as related to the acquisition of information, and its analysis and delivery in a very short time; modularity, or the flexibility to integrate or adapt modules; a high level of access; and fluid and accessible knowledge management. It can be seen that all these features are adopted and applied by the majority of online services through applications which are downloaded by the customer. These features can facilitate the purchasing process and business operations. The objective in this study was to focus especially on CE, and as mentioned below, this concept is cultivated through a cycle, the key success factor of which is trust and loyalty. It is suggested here that adequate information in adequate time and fluid information is necessary.

In the same vein, IA can generate a high quantity and quality of information, which can be saved and processed with high frequency, delivered in real-time with a breadth of variety and modulated in a simple manner (Tiago et al., 2019).

Becoming more familiar with IA makes its use easier, with consumers growing more likely to trust it and adopt it over the long term (Howard & Borenstein, 2020). In this context, IT culture as defined by Walsh (2014) can offer a clear idea of IT adoption. Based on this theory, the use and adoption of technologies depends on an individual's social practices, which determine preferences related to personal needs and motivation (Abubakre et al., 2017).

This means, supposing that the use of digital tools can stimulate engagement of the consumer (De Ruyter et al., 2019; Leek et al., 2017), it must be considered that this effect requires some pre-conditions, and is not an automatically successful process. To stimulate CE through AI, the preferences of the consumer must adapt to the idea of the adoption of AI and trust in IA if used. This pre-supposes that the customer must have motivations and personal need to adopt IA. Frey (2020) adds that the use and adoption of AI requires confidence.

So, trust and confidence in the use of IA must be built to achieve customer engagement. Motivations and personal needs can be defined by the customer, thus establishing their preferences, but the more important question here is how these important criteria can then be communicated to the service provider. A communication process must be established and co-interaction between a customer and the service provider must be created, but the most significant element remains the credibility of information collected and communicated, to achieve a high level of trust. Remembering that CE is based on social, emotional, cognitive and behavioral investment, coherence between these factors also has to be maintained. The multidimensionality of this concept requires a dynamic, co-constructive model.

Due to the absence of direct contact, information collected via IA must be reliable, valid and precise. Günther et al. (2017) argue that where data are not collected at the right time, or with precision and reliability, this can lead to an inappropriate decision being taken. This decision, from the perspective of this paper, can negatively affect the perceptions of the customer with regard to a service, and the impact of this grows increasingly significant in this case due to the absence of any human interaction and direct discussion which could provide a surer channel for clarifying a misunderstanding if one exists. Added to this, this dissatisfaction can be transmitted to other consumers via comments on applications, for example as in a review, which may be visible to all users and can consequently affect the image of the service provider. Human biases continue to exist, and there has not been a reliable or worthy algorithm created to the present which can provide a high level of successful automatic decision-making (Davenport, 2020). However, the use of AI can produce greater opportunity to make a repetitive and complex task more efficient and low-cost (Wyman, 2017).

### Mutual trust, intelligent automation and customer engagement

Sabel (1993) argues that trust concerns the establishment of mutual confidence between different partners in a transaction. In fact, trust helps different parties to disclose their personal and unique point of view of a specific problem to facilitate its effective understanding (Mattessich et al., 2001).

Panchapakesan et al. (2017) insist on the importance of communication and the two-way exchange of information to generate trust and commitment. In other words, according to these authors, the level of trust depends on the quality and importance of the communication. This observation leads to the supposition that IA, with the features and specifics discussed below, is a vector of exchange and communication and thus can increase trust.

From the perspective of social exchange, trust is not related only to the person, and can be defined through the experience occurring between two parties on a level of fairness and kindness which depends on social factors (Zhang & Jia, 2010). This means that trust is limited by external factors related to the nature of the relationship itself.

The kind of trust defined here is based on the nature of the parties to it. In fact, it is different from the organizational trust which can be defined inside organizations (Schoorman et al., 2007) or the interpersonal trust between persons, groups and organizations (Hurley, 2006, 2012). This mutual trust depends instead on the existence of trust between person and organization (or service provider) over a long time period.

This relationship of trust can be facilitated through the name (brand) or the service offered. Further, it is assumed that there is ‘tangible and intangible trust’. In other words, ‘tangible’ trust can be generated through the service itself, but intangible trust remains related to the feeling of the person or consumer of the level of satisfaction of their need.

To determine the antecedents of this specific type of trust, which is represented by a mix of interpersonal and organizational trust, it is necessary to review antecedents for each category, to ultimately define the list of antecedents adopted here.

Interpersonal trust depends on attitudes, motivations, emotions and personality (Christie et al., 2015; Sherwood & DePaolo, 2005). Meanwhile, organizational trust is based on ethics, shared values, socio-cultural context (Das & Teng, 2001) and open communication, as well as participation (Pucetaite et al., 2010). All of these aspects seem to be more important in the service context (Agariya & Singh, 2011).

To be both more pragmatic and dynamic, a tripartite perspective is adopted, integrating customer, interpersonal trust and organizational trust (Poon et al., 2017; Yasir & Majid, 2017).

Organizational trust, in this view, supposes that staff, considered as an interface in the relation exchange, have an impact on the willingness of the consumer to maintain this relationship over a longer time (Weitz & Bradford, 1999). This type of trust is associated with relational behavior (Palmer & Bejou, 1994), and this last variable has been approached through a great number of dimensions. Considering the needs of this research however, three main dimensions are adopted which satisfy those needs: cooperative behavior, listening behavior and sharing of information.

Communication, as ensured by AI and IA, has a positive impact on trust, and this effect has been tested by many researchers (Ball et al., 2004; Garbarino & Johnson, 1999). Further, Blackston (1992) argues that communication helps the generation of trust in the relationship between customer and service provider, and Morgan and Hunt (1994) consider it to be a determinant factor in the development of trust. Moreover, if customers do not face difficulties in understanding and obtaining information by means of adequate communication, this will facilitate equilibrium in the partnership, and it will be easy for the parties to understand each other (Ball et al., 2004). Gatfaoui (2000) demonstrates that successful communication can provide and maintain loyal customers.

### The emergent conceptual framework

The conceptual framework presented in this research is based on a dynamic approach to CE. It is a co-construction model which expresses a bi-directional relationship, and represents an integrative model between AI, CE and mutual trust (MT). The major hypothesis adopted here supposes that CE is developed through a process, and that the most important state in this process is related to the development of mutual trust, because this supposes an exchange of trust between customer and service provider. The customer must trust the service provider in terms of quality, satisfaction and reliability, for example. The service provider trusts the customer to express their dissatisfaction and report problems to find the appropriate solutions, while remaining faithful in use of the provider’s service. During this process, corporate communication seems to be important in facilitating the exchange of information, and artificial intelligence, by the definition of automation intelligence, is the best way to assist and ensure the continuity of exchange of information in both directions. Added to this, trust is omnipresent during the development of this process and must be defined on an interpersonal and organizational level to ensure coherence and continuity.

**Fig. 1:**
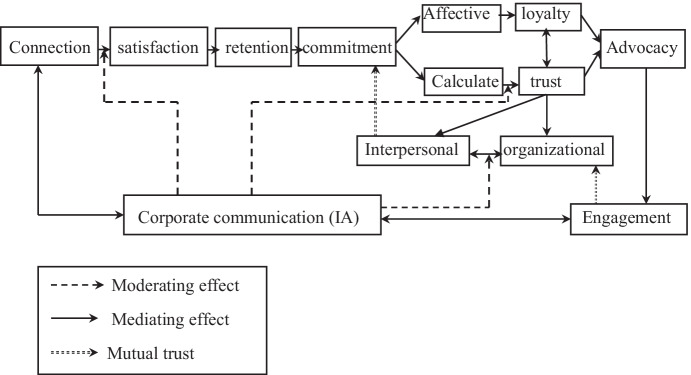
Dynamic approach of a conceptual model

As shown in Fig. 1, IA assists and defines the corporate communication process. In this model, IA can be considered as a mediating—moderating effect to trigger mutual trust. Two main pathways for this can be distinguished:

The first pathway flows from customer to service provider in order to develop CE. IA ensures a moderating role twice during this process. It is assumed here that a high level of satisfaction depends on the nature and the reliability of the information provided after connection. A high level of satisfaction is associated with an intensive exchange of good information. The link between commitment (affective and calculative) and trust will then be moderated by IA.

The second pathway is represented by the service provider and customer. In this case, IA will be a mediating variable which can link to the CE generated with the connection (the first step in this process). The existence of IA will make the exchange easier, and so the response of the service provider to the customer problem or dissatisfaction will be faster. Ultimately, the development of a long-term relationship will depend on the development of mutual trust between CE, organizational trust and interpersonal trust, and commitment.

To make this pathway more useful, a matrix is presented which integrates the CE process or stages and perspectives on CE with relative importance in this research: AI and mutual trust. It must be remembered that AI is understood through specific tools in this field of research on intelligent automation.

Engagement is entirely related to mutual trust, and the construction of this trust depends on the exchange of information through AI.

In order to make this conception more operational, we will proceed to a quantitative analysis which is detailed in the next section related to the methodology. Results and discussion will be detailed too. Here, the critical pathway represented in Fig. 1 is developed and applied in order to operationalize our main objective and identify its main aspects. This is an empirical approach to the objective as specified below. In other words, this is a theoretical process which must be adopted to develop mutual trust through corporate communication. To make this one more operational, it seems more appropriate at this stage to verify, empirically, if these relationships really exist. Thus, it becomes necessary to test the interrelation between basic variables: corporate communication, consumer engagement and mutual trust.

## Methodology

**Fig. 2:**
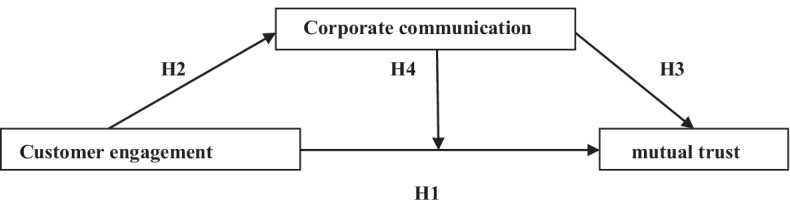
Conceptual framework

Based on the previous analysis, the study aims to test effects and relationships between three main concepts; customer engagement, mutual trust and corporate communication (Fig. 2).

In this case, four main hypotheses were adopted according to which the mediate—moderate effect of corporate communication will be tested:

H1: Customer engagement positively affects mutual trust;
H2: Customer engagement positively affects corporate communication;
H3: Corporate communication positively affects mutual trust; and
H4: Corporate communication affects the link between customer engagement and mutual trust.

In the next section, the methodology used is detailed, identifying the support used to collect data, items collected to construct this support and the sample. After this, the results are presented and interpreted, and a discussion follows.

### Methodology

The methodology adopted here is based on a quantitative approach through a questionnaire conceived and administered with 330 consumers of different restaurants on the region of Riyadh in Saudi Arabia. As mentioned at the beginning of this paper, this field was selected as of interest because all people in this pandemic period are worried about their health to and staying safe.

### Sample

To collect data, a survey was conducted with restaurant consumers through an online self-administered questionnaire using Google Forms. In all, 350 questionnaires were distributed, but only 330 of those could be utilized. Participants were asked to indicate their agreement or disagreement with propositions according to a five-point Likert scale. Items used here are detailed with references in Table 1. The sample is composed of 60.6% female respondents, aged between 18 and 35. The remainder is composed of male respondents, most of whom are aged between 15 and 40. The questionnaire was presented in four sections: the first is related to the demographic’s characteristics; the second talks about consumer engagement; the third part regards mutual trust; and the last part contains by items about corporate communication.

**Table 1:** CE matrix

	Connection	Satisfaction	Retention	Commitment	Advocacy	Engagement
Social	AI	T	T	T/AI	T	T
Emotional	T	T	T	T	T	T
Cognitive	AI	AI	AI	AI	AI/T	T
Behavioral	AI	AI/T	AI	AI	AI	T

Responses were coded and analysed using SPSS 16.0, to identify robustness and the correlation between variables. It should be remembered that this research is focused on the testing of relations in order to adopt an innovative framework for mutual trust and consumer engagement.

To make the measure of different constructs measurable and operational, it is necessary to define for each construct a number of items mentioned in previous research. Table 2 provides details related to its construct on term of number of items and references.

**Table 2:** Dimensions and items

Construct	Number of items	References
Mutual trust	3 items	Bagozzi and Dholakia (2006) Cummings and Bromiley (1996)
Customer engagement	16 items	Cheung et al. (2011)
Corporate communication	22 items	Guofeng and al. (2020)

#### Data collection

As a quantitative study, a questionnaire was adopted to collect data. This was divided into three main parts, related to the three main variables, in line with the order in the theoretical framework: the first is related to customer engagement through three dimensions: cognitive, behavioral and emotional. The second is related to corporate communication, represented by four dimensions: network centrality, network scale, relationship strength and relationship stability Finally, the third part represents mutual trust as a result of this interaction, which is important for the theoretical process presented in the first part of this paper.

Items for these variables, along with references, are presented in Table 2. This questionnaire will be administered among the food and restaurant sector because, in the current circumstances, organizations have to perform and create new ways to deliver their services and protect consumers at the same time, due to the social distancing imposed by the COVID 19 pandemic and its dangerous impacts on human health.

## Results and discussion

A component analysis was performed in order to test the multidimensionality or one-dimensionality of the constructs. Table 2 shows factor loading and the Cronbach’s alpha for its group of items. It is clear from this that corporate communication (four dimensions) and customer engagement (three dimensions) seem to be multidimensional.

As a multidimensional concept, corporate communication is composed of four dimensions: Network centrality; Network scale; Relationship strength; and Relationship stability. This can be explained by the diversity of technologies and tools used in this context.

Customer engagement is composed of cognitive, emotional and behavioral aspects (Cheung et al., 2011). In fact, this analysis confirms the previous analysis in which customer engagement was considered as a constructive concept. There are levels to achieving customer engagement, and this idea was detailed in the first part of this paper through the process detailed in Fig. 1.

Discriminant validity is used to examine the degree of distinction between different measures of concepts (Bagozzi, 2006) and is also understood through the AVE. All values are accepted and the items used are coherent for two models, i.e., the model related to the mediating effect and that for the moderating effect of corporate communication developed and reinforced by artificial intelligence techniques, as explained in the last section. Table 3 synthesizes the main results related to this exploratory step.

**Table 3:** Component analysis

Constructs	Dimensions	Items	Loading	Var (%)	Cronbach’s alpha
Mutual trust	Mutual trust	MT1 MT2 MT3	0.56 0.63 0.66	67	0.78
Customer engagement	Cognitive	C1 C2 C3 C4 C5	0.53 0.63 0.42 0.55 0.64	27	0.81
	Emotional	E1 E2 E3 E4 E5	0.78 0.71 0.69 0.64 0.67	15	0.77
	Behavioral	B1 B2 B3 B4 B5 B6	0.55 0.57 0.54 0.62 0.58 0.64	12	0.73
Corporate communication	Network centrality	NC1 NC2 NC3	0.47 0.40 0.32	9	0.55
	Network scale	NS1 NS2 NS3 NS4 NS5	0.43 0.57 0.59 0.41 0.51	8	0.59
	Relationship strength	RS1 RS2 RS3 RS4 RS5	0.66 0.69 0.58 0.53 0.59	21	0.72
	Reciprocity	R1 R2 R3 R4	0.71 0.64 0.66 0.63	23	0.81

The cognitive dimensions in the sample represent the majority of information collected, and this means that the process related to customer engagement is mainly constructed based on a reflexive approach based on information collected through corporate communication. Behavioral and emotional aspects have the same importance, and it can be assumed that these levels depend on the relative importance of the first step: cognitive.

For corporate communication, reciprocity and relationship strength are significant, while the remaining dimensions seem to be non-representative in this case.

A descriptive statistic related to the construct is detailed in Table 4 to facilitate the correlation test. Results of correlation (summarized in Table 5) between the different concepts (shown in Table 5) demonstrate that consumer engagement is correlated with corporate communication (*β* = 0.6) more than mutual trust (*β* = 0.4). This can be explained by the consumer’s need for information to make a decision and ensuring feedback if necessary.

**Table 4:** Descriptive statistics

	Mean	Std. Deviation	*N*
Mutual trust	3.76	2.025	498
Corporate communication	3.63	1.777	484
Customer engagement	3.77	1.961	469

**Table 5:** Correlation test

	Customer engagement	Mutual trust	Corporate communication
Customer engagement			
Pearson correlation	1	0.421**	0.619**
Sig. (2-tailed)		0.000	0.000
Covariance	4.102	0.869	0.867
*N*	330	330	330
Mutual trust			
Pearson correlation	0.242**	1	0.298**
Sig. (2-tailed)	0.000		0.000
Covariance	0.869	3.157	1.046
*N*	330	330	330
Corporate communication			
Pearson correlation	0.219**	0.298**	1
Sig. (2-tailed)	0.000	0.000	
Covariance	0.867	1.046	3.847
*N*	330	330	330

**Correlation is significant at the 0.01 level (2-tailed)

For the indirect relationship between customer engagement and mutual trust, the mediating effect of corporate communication is admitted. Indirect effect is more important with the presence of corporate communication, and the correlated effect of corporate communication and mutual trust is significant. In order to check the direct relationship of customer engagement and mutual trust, a model without corporate communication was run. The coefficient of correlation is about 0.3 but it is still significant, and for this reason, the hypothesis of moderating effect was adopted. This strong indirect effect of customer engagement on mutual trust is logically admitted due to the existence of a great number of products and high levels of competitiveness. Customers need increasing amounts of information in order to make adequate and reasonable choices, and if satisfied, the customer becomes habitual and the process of mutual progress supported by the mutual trust is defined.

This interaction between customer engagement and mutual trust is calculated in order to facilitate understandings of corporate communication here.

## Discussion and implications

Artificial intelligence techniques have considerably changed features of communication between consumer and service provider. In this sense, these technologies assist in the development of corporate communication in which the consumer faces a great number of messages at the same time. For this reason, the consumer has moved from being a passive receiver to an active agent able to compare and negotiate conversations with service providers in terms of quality, price and privileges.

Fast-evolving technologies and artificial intelligence mean that markets and their dynamics must be changed: organizations must revisit their communications strategy, and it is not enough to produce or to assist the consumer with simple engagement: there is a need to gain mutual trust for the long interaction and stable relationship on which the organization’s survival depends. In fact, corporate communication can be considered at the same time as opportunity, and the development of effective communication strategies have become the priority.

With reference to the existing literature on consumer engagement, this study investigates another side to or developed version of this concept, which is promoted to a mutual trust which can be of benefit for both consumer and organization. This mutual trust can be assisted and stimulated by use of corporate communication via artificial intelligence techniques. In this case, the communication process becomes more transparent: the consumer can maximize their satisfaction and the organization can guarantee its survival as directly related to consumer satisfaction levels. It can be stated that mutual trust generates mutual satisfaction and mutual survival in terms of the need for satisfaction for the consumer and profit for the organization.

Moreover, this research has empirically tested empirically the constructed model that places corporate communication between consumer engagement and mutual trust. The results confirm all hypotheses defined here, and this supports the dynamic approach adopted in the first part of this research. It is important to remember here that the result suggest that the link between consumer engagement and mutual trust is indirect. This means that the transition between these concepts is not automatic, but must be assisted and pushed**.**

The mediating-moderating effect is verified and admitted, and indirect relationships are quantified. However, the implicit composition of the construct must be revisited. There is a great difference between dimensions in the same construct: in other words, the research supports the active role of the customer in stimulating their own engagement and reinforcing the reciprocity with the service provider. The ultimate objective is to make this mutual trust the origin of a co-constructive process which is win–win. The progressive and processual approach defined in the first conceptual framework needs to be detailed and enriched by other variables: especially noting the differentiated effect between dimensions, as mentioned below. Interestingly, advances related to artificial intelligence can facilitate this interactive approach, but further research must be aware that the time taken to construct this long-time relationship becomes determinant in itself, and it is necessary to look for mechanisms and tools to reduce that time as far as possible. The digital arena facilitates such interactive marketing efforts on a large scale. The findings of this research support the existing literature about customer engagement and artificial intelligence. This study is important because it takes consideration of this important area and offers some directives to promote and understand how AI can affect markets and consumer behavior. This will help orient managers to recognize and identify adequate communication via the customer to maximize the mutual trust which is crucial for the success of any service or brand.

However, some limitations are admitted in this context, because customer engagement is understood via a limited number of communication tools, and there is a need to enlarge tools, approaches and methods of communication. Although methodology used here aims to explore relationships, their significance and effects, more appropriate techniques can be adopted to test the conceptual framework with a higher number of respondents.

## Conclusion

This paper is an attempt to understand CE development in order to provide an operationalized approach to this concept. The theoretical model developed at the end of this research can guide service providers in the development of CE through IA and mutual trust.

It represents a dynamic approach with a co-constructive perspective in which the customer is considered as an active and reactive actor. The model includes different stages adopted in the development of CE. The main foundation for CE continuity here is mutual trust—related exclusively to an affective commitment and ensured by AI applied to build a successful information exchange. Here, the features and specificities of AI and IA have been detailed to clarify how this process can operate.

The analysis confirms, also, the multidimensionality of CE, and this explains the need to develop a dynamic, co-constructive model.

The literature review reveals that there are specific activities and tactics which can be applied to generate CE, stimulate it and optimize it. Also, there are antecedents and challenges related to a successful CE. By successful, a long-term relationship between customer and service provider is meant.

This model can be tested and adapted in further research in order to quantify different links. Also, it would be more interesting if the model could be reinforced by the integration of specific determinants of mutual trust, and specify what kind of AI can be adopted. In other words, an instrumental approach to this model could make it more pragmatic.

## Data Availability

Data are available if required.

## Appendix: Questionnaire items

### Mutual trust

Our firm has strong confidence that our customers will provide the best advice in regard to our businesses and for our sake.

Our firm is able to provide sincere aid to our customers.

Our customers keep their words to our firm.

### Customer engagement

#### Cognitive engagement

I am interested in anything about the product.

Using this product makes me forget about everything else.

I find it difficult to detach myself from this product.

My mind is focused when using this product.

I pay a lot of attention to this product.

#### Emotional engagement

I am very enthusiastic about this product.

This product inspires me.

This product means a lot more to me than other brands.

I am excited when using this product.

Time flies when I am using this product.

I am proud of using this product.

#### Behavioral engagement

I would continue using this product for a very long time.

I feel more alive when using this product.

Over time, this product becomes more important to me.

I would continue using this product even when things do not go well.

I try my hardest to make my relationship with X work.

### Corporate communication

There are guidelines about the format of messages, typology and how colors will be used; standardization is achieved in this respect.

Rules and regulations about communication exist in this corporation and they are in effect.

The responsibilities and authority of employees in communications departments are clearly defined.

Corporate image is an issue which is emphasized.

Corporate identity activities are given importance in this corporation.

Corporate vision is clear.

Media relations are carried out successfully.

Corporate mission is defined clearly.

Brand values of the corporation are defined clearly.

Investor relations are carried out successfully.

Corporate philosophy is defined clearly.

If our communications departments were asked to name the characteristics that differentiate your corporation from others, they would give overlapping answers.

Marketing activities are carried out successfully.

Management communication is carried out successfully.

Corporate advertising is carried out successfully.

Internal communication is carried out successfully.

These brand values are adopted by employees.

The corporation embodies the value it gives to its employees through activities it organizes for them.

Corporate employees try to achieve the objectives defined in the corporate vision.

## Abbreviations

CE: Customer engagement
IA: Intelligent automation
MT: Mutual trust
AI: Artificial intelligence
SCA: Sustainable competitive advantage
AI: Artificial intelligence
T: Trust
